# Interstitial cells in calcified aortic valves have reduced differentiation potential and stem cell-like properties

**DOI:** 10.1038/s41598-019-49016-0

**Published:** 2019-09-10

**Authors:** Maria Bogdanova, Arsenii Zabirnyk, Anna Malashicheva, Katarina Zihlavnikova Enayati, Tommy Aleksander Karlsen, Mari-Liis Kaljusto, John-Peder Escobar Kvitting, Erik Dissen, Gareth John Sullivan, Anna Kostareva, Kåre-Olav Stensløkken, Arkady Rutkovskiy, Jarle Vaage

**Affiliations:** 1 Department of Molecular Medicine, Institute of Basic Medical Sciences, University of Oslo, Oslo, Norway; 2 Almazov National Medical Research Centre, Saint Petersburg, Russia; 3 Institute of Cytology, Russian Academy of Sciences, Saint Petersburg, Russia; 4 Saint Petersburg State University, Saint Petersburg, Russia; 5 Norwegian Center for Stem Cell Research, Department of Immunology, Oslo University Hospital, Oslo, Norway; 6 Department of Cardiothoracic Surgery, Oslo University Hospital, Oslo, Norway; 7 Institute of Clinical Medicine, University of Oslo, Oslo, Norway; 8 Institute of Immunology, Oslo University Hospital, Oslo, Norway; 9 Hybrid Technology Hub - Centre of Excellence, Institute of Basic Medical Sciences, University of Oslo, Oslo, Norway; 10 Department of Pediatric Research, Oslo University Hospital, Oslo, Norway; 11 Department of Woman and Children Health, Karolinska Institute, Stockholm, Sweden; 12 Center for Heart Failure Research, Oslo University Hospital, Oslo, Norway; 13 Department of Cardiology, Akershus University Hospital, Lørenskog, Norway; 14 Department of Emergency and Critical Care, Oslo University Hospital, Oslo, Norway

**Keywords:** Mechanisms of disease, Valvular disease

## Abstract

Valve interstitial cells (VICs) are crucial in the development of calcific aortic valve disease. The purpose of the present investigation was to compare the phenotype, differentiation potential and stem cell-like properties of cells from calcified and healthy aortic valves. VICs were isolated from human healthy and calcified aortic valves. Calcification was induced with osteogenic medium. Unlike VICs from healthy valves, VICs from calcified valves cultured without osteogenic medium stained positively for calcium deposits with Alizarin Red confirming their calcific phenotype. Stimulation of VICs from calcified valves with osteogenic medium increased calcification (p = 0.02), but not significantly different from healthy VICs. When stimulated with myofibroblastic medium, VICs from calcified valves had lower expression of myofibroblastic markers, measured by flow cytometry and RT-qPCR, compared to healthy VICs. Contraction of collagen gel (a measure of myofibroblastic activity) was attenuated in cells from calcified valves (p = 0.04). Moreover, VICs from calcified valves, unlike cells from healthy valves had lower potential to differentiate into adipogenic pathway and lower expression of stem cell-associated markers CD106 (p = 0.04) and aldehyde dehydrogenase (p = 0.04). In conclusion, VICs from calcified aortic have reduced multipotency compared to cells from healthy valves, which should be considered when investigating possible medical treatments of aortic valve calcification.

## Introduction

Calcific aortic valve disease is one of the leading causes of cardiovascular mortality^1^. The aortic valve is most prone for calcification and in need of surgical treatment^2^. Although minimally invasive catheter-based techniques are gaining volume, open heart surgery with aortic valve replacement is still the golden standard. Heart valve prostheses are either mechanical or biological; with both types having their inherent side effects and limitations. Consequently, implantation of heart valve prostheses has been characterized as “replacing one disease with another”. The strongest risk factors for calcific aortic valve disease are advanced age and bicuspid aortic valve^3–7^. Thus, calcific valve disease is expected to represent an increasing burden to health care due to an ageing population^8^.

Heart valve calcification is an active process with similarities to both bone formation and atherosclerosis^9^. Valve interstitial cells (VICs) are crucial in the calcification process and may be the key to understand the mechanisms of heart valve calcification^7^. VICs have a mesenchymal origin, and they are able to differentiate *in vitro* into osteogenic, adipogenic, chondrogenic, and myofibroblastic lineages^10^. The progression of the disease involves inflammation, oxidative/mechanical stress, fibrosis, and finally calcification^4–7,11,12^. VICs may develop into either preosteoblasts or myofibroblasts^7^, altering the physical and anatomical properties of the valve. In the latter case, the cells form multicellular aggregates (nodules), which undergo apoptosis leading to the formation of apoptotic bodies and serving as nucleation points for calcium crystals with deposition of hydroxyapatite^13^. At this stage the process enters a self-perpetuating propagation phase^11^.

In order to develop new therapeutic agents that slow, stop, or even reverse the calcification process in valve leaflets, it is necessary to understand the histological and cellular changes that occur during the disease^14^. Particularly, it is interesting to know whether the pathological processes have a potential to be reversed. The purpose of the present study was to compare the phenotype and the potential of VICs from calcified and healthy aortic valves to differentiate into different cell lineages as well as to evaluate their proliferative activity and degree of “stemness”.

## Results

### Cells from calcified valves have osteogenic phenotype

**Figure 1:**
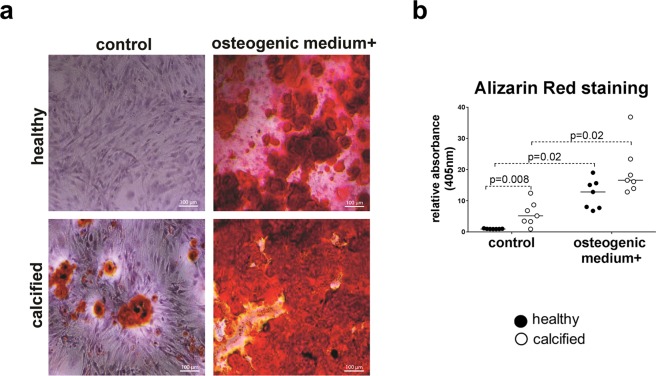
(**a**) Microscopic visualization (10 x objective) of calcification by Alizarin Red staining of interstitial cells isolated from healthy (n = 7) and calcified (n = 7) aortic valves and cultured for 21 days in standard growth medium (control) or osteogenic medium, as indicated. **(b)** Quantification of Alizarin Red staining by absorbance at 405 nm. Groups were compared by Wilcoxon matched-pairs signed rank test (control vs osteogenic medium+) or Kolmogorov-Smirnov test (healthy vs calcified). Lines in scatter plots represent the median.

To investigate the ability of VICs to calcify, we stimulated cells for 21 days with osteogenic medium. VICs from calcified valves, but not from healthy valves, accumulated calcified nodules even in standard growth medium without stimulation with osteogenic medium (Fig. 1a,b). After stimulation with osteogenic medium there was no statistically significant difference in calcification between the sample groups (Fig. 1b).

### Gene expression in valve interstitial cells after osteogenic stimulation

**Figure 2:**
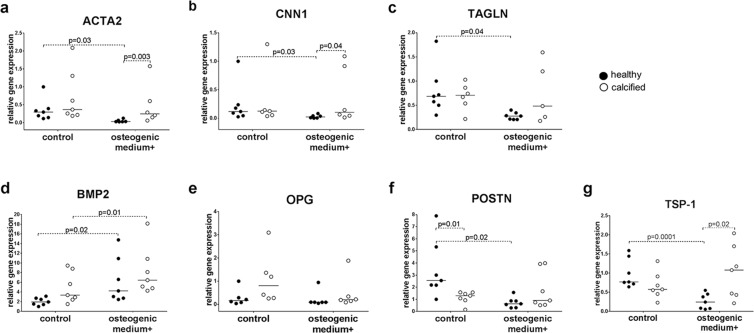
Relative gene expression, as measured by quantitative reverse transcription PCR, of calcification- and myofibroblast-related genes: **(a)** *ACTA2* (alpha–smooth muscle actin 2), **(b)** *CNN1* (calponin), **(c)** *TAGLN* (transgelin), **(d)** *BMP2* (bone morphogenetic protein 2), **(e)** *OPG* (osteoprotegrin), **(f)** *POSTN* (periostin) and **(g)** *TSP-1* (thrombospondin 1) in interstitial cells isolated from healthy (n = 6–7) or calcified (n = 5–7) aortic valves and cultured for 21 days in standard growth medium (control) or osteogenic medium. Groups were compared by Student’s t-test (parametric) or Wilcoxon matched-pairs signed rank test (non-parametric) for paired data (control vs osteogenic medium+) and unpaired Student’s t-test (parametric) or Mann-Whitney test (non-parametric) for unpaired data (healthy vs calcified). Lines in scatter plots represent the median.

To investigate the potential of VICs from healthy and calcified valves to differentiate into osteoblasts after 21 days of stimulation with osteogenic medium, we analyzed the expression of calcification-related genes: *BMP2* (bone morphogenetic protein 2), *OPG* (osteoprotegrin)^15^, *POSTN* (periostin)^16^ and *TSP-1* (thrombospondin 1)^17^, as well as myofibroblast-related genes: *ACTA2* (alpha-smooth muscle actin 2), *CNN1* (calponin) and *TAGLN* (transgelin)^18^ by RT-qPCR. We observed no differences in the expression of all the genes selected for analysis, for undifferentiated cells from both healthy and calcified aortic valves except for *POSTN* (Fig. 2). Undifferentiated VICs from healthy valves had higher expression of *POSTN* gene as compared to VICs from calcified valves (Fig. 2f). After osteogenic differentiation, expression of the myofibroblastic markers (*ACTA2*, *CNN1* and *TAGLN)* decreased in VICs from healthy aortic valves, but did not change in calcified valves (Fig. 2a,b,c). The expression of *ACTA2* and *CNN1* was higher in cells from calcified valves after stimulation with osteogenic medium (Fig. 2a,b).

Cells from both healthy and calcified valves had increased expression of osteogenic marker *BMP2* after stimulation with osteogenic medium (Fig. 2d), whereas *OPG*, did not increase (Fig. 2e). The expression of *POSTN* and *TSP-1* was downregulated in differentiated VICs from healthy valves, but did not change in cells from calcified valves (Fig. 2f,g). After osteogenic differentiation *TSP-1* expression was higher in VICs from calcified valves compared to cells from healthy valves (Fig. 2g).

Collectively our data suggest that osteogenic stimulation reciprocally inhibits myofibroblastic pathway in healthy VICs, but not in calcified ones.

### Differentiated cells from healthy valves have higher expression of myofibroblastic genes

**Figure 3:**
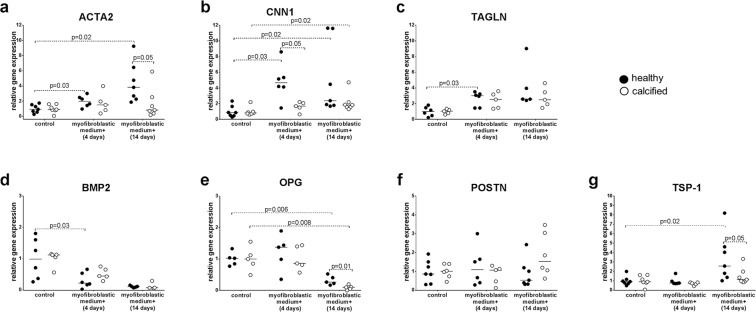
Relative gene expression, as measured by quantitative reverse transcription PCR, of calcification- and myofibroblast-related genes: **(a)** *ACTA2* (alpha–smooth muscle actin 2), **(b)** *CNN1* (calponin), **(c)** *TAGLN* (transgelin), **(d)** *BMP2* (bone morphogenetic protein 2), **(e)** *OPG* (osteoprotegrin), **(f)** *POSTN* (periostin) and **(g)** *TSP-1* (thrombospondin 1) in interstitial cells isolated from healthy (n = 5–7) or calcified (n = 5–7) aortic valves and cultured for 4 and 14 days in standard growth medium (control) or myofibroblastic medium. Groups were compared by Student’s t-test (parametric) or Wilcoxon matched-pairs signed rank test (non-parametric) for paired data (control vs myofibroblastic medium+) and unpaired Student’s t-test (parametric) or Mann-Whitney test (non-parametric) for unpaired data (healthy vs calcified). Lines in scatter plots represent the median.

Data for myofibroblastic differentiation were normalized to control group without differentiation harvested at 4 and 14 days respectively. Raw data are presented in Supplementary Fig. S1. Only cells from healthy aortic valves increased expression of myofibroblastic markers: *ACTA2*, *CNN1* and *TAGLN* after stimulation with myofibroblastic medium for four days (Fig. 3a,b,c). After 14 days of differentiation, the expression of *CNN1* increased in cells from both healthy and calcified valves, while expression of *ACTA2* was upregulated only in cells from healthy valves. There were significant differences in higher expression of *ACTA2* (14 days) and *CNN1* (4 days) in cells from healthy valves compared to calcified (Fig. 3a,b).

Stimulation with myofibroblastic medium tended to decrease the expression of *BMP2* in cells from both healthy and calcified valves (Fig. 3d). The expression of *OPG* was significantly downregulated after 14 days of stimulation of cells from both healthy and calcified valves (Fig. 3e). The expression of *POSTN* did not change in cells from either healthy or calcified valves (Fig. 3f). *TSP-1* was increased in VICs from healthy valves after stimulation with myofibroblastic medium for 14 days compared to differentiated cells from calcified valves (Fig. 3g).

These data indicate that cells from calcified valves have reduced potential to differentiate into myofibroblasts, compared to cells from healthy valves. Osteogenic pathway is reciprocally downregulated in both cells from calcified and healthy aortic valves during myofibroblastic differentiation.

### Differentiated cells from healthy valves have higher expression of myofibroblast marker proteins and a greater ability to contract

**Figure 4:**
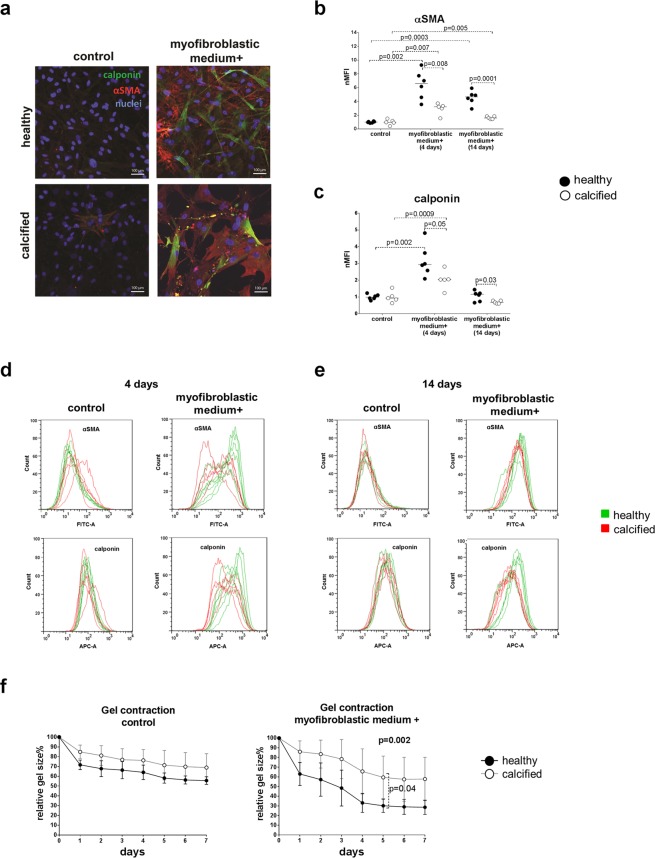
(**a**) Representative pictures (10 x objective) of immunofluorescence staining for myofibroblastic markers: alpha-smooth muscle actin (αSMA) and calponin in interstitial cells isolated from healthy (n = 6) and calcified (n = 5) aortic valves and cultured for 14 days in standard growth medium (control) or myofibroblastic medium. αSMA (red), calponin (green), cell nuclei (Hoechst 3342/blue). **(b–e)** Flow cytometry analysis of αSMA and calponin expression in interstitial cells isolated from healthy (n = 6) or calcified (n = 5) aortic valves and cultured for 4 or 14 days with standard medium (control) or myofibroblastic medium. **(b**,**c)** Data are presented as normalized mean fluorescence intensity (nMFI). Groups were compared by Student’s t-test for paired data (control vs myofibroblastic medium+) and unpaired Student’s t-test for unpaired data (healthy vs calcified). Lines in scatter plots represent the median. **(d**,**e)** Data are presented as histogram overlays of individual samples from healthy (green curves) or calcified aortic valves (red curves). **(f)** Comparison of collagen gel contraction between interstitial cells isolated from healthy (n = 4) and calcified (n = 4) aortic valves cultured in control or myofibroblastic medium over a period of seven days. Data are presented as measurements of relative gel sizes in percent. Gel sizes on day 0 were considered as 100%. Data were analyzed by two-way ANOVA with repeated measures. Differences between healthy and calcified donors were determined with Sidak’s multiple comparison post-test significant p-value is shown on 5^th^ day of measurement. Values are expressed as mean ± SD. Overall p-value from two-way ANOVA is shown in bold.

VICs from both healthy and calcified valves increased the expression of the myofibroblastic markers αSMA and calponin after 14 days of culture in myofibroblastic medium (Fig. 4a). To obtain more quantitative data, VICs were analyzed by flow cytometry after 4 or 14 days of culture in either myofibroblastic medium or standard growth medium (Fig. 4b,c,d,e). Firstly, the expression of αSMA and calponin correlated among individual cells, as demonstrated by two-color flow cytometry, in accordance with the expectation that expression of these two markers is co-regulated (Supplementary Fig. S2). Individual cells were discretely distributed between both αSMA^−^/calponin^low^ population (undifferentiated VICs from healthy valves) and αSMA^+^/calponin^bright^ population (differentiated VICs from healthy valves) (Supplementary Fig. S3). Together, the present data support the idea that αSMA^+^/calponin^bright^ population represents truly differentiated fraction of VICs.

After 4 days in myofibroblastic medium, VICs from both healthy and calcified valves displayed heterogeneity regarding to αSMA and calponin expression. VIC populations from healthy valves contained a higher fraction of αSMA^+^/calponin^bright^ cells indicating more prominent myofibroblastic differentiation compared to VIC populations from calcified valves (Fig. 4d). No difference was seen between the groups when cultured in standard medium (Fig. 4d). After 14 days of culture in myofibroblastic medium, both sample groups attained more homogeneous distribution, containing mostly αSMA^+^/calponin^bright^ cells (Fig. 4e). The αSMA^+^/calponin^bright^ cells from healthy valves expressed both αSMA and calponin at slightly higher levels compared to cells from calcified valves (Fig. 4e). The quantification of flow cytometry data following normalization by mean fluorescence intensity (nMFI) showed that nMFI cells from healthy valves stained for αSMA and calponin was higher compared to cells from calcified valves at both time points (Fig. 4b,c).

To evaluate myofibroblastic differentiation functionally, VICs from healthy or calcified valves were analyzed in a gel contraction assay. In standard medium, we did not observe significant differences in gel contraction between VICs from healthy and calcified valves. However, upon stimulation with myofibroblastic medium the cells from healthy valves contracted faster and stronger than cells from calcified valves. On the fifth day of stimulation by myofibroblastic medium the mean gel size with cells from healthy valves was reduced by 70% compared to 40% for cells from calcified valves (Fig. 4f).

These data confirm that VICs from calcified valves differentiate slower into myofibroblasts and possess lower potential to myofibroblastic differentiation compared to cells from healthy valves.

### Cells from healthy and calcified valves have equal potential to differentiate down the chondrogenic pathway

Both cell pellets from healthy and calcified aortic valves as well as bone morrow mesenchymal stem cells (BM-MSCs) after stimulation with chondrogenic medium for 21 days were stained positively for Alcian Blue indicating the presence of proteoglycans (marker of chondrocytes) compared to cells cultured in standard growth medium (Supplementary Fig. S4).

**Figure 5:**
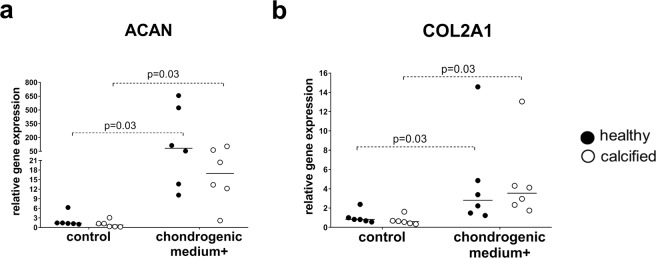
Relative gene expression, as measured by quantitative reverse transcription PCR, of chondrogenic markers: **(a)** *ACAN* (aggrecan), **(b)** *COL2A1* (collagen type II alpha 1 chain), in interstitial cells isolated from healthy (n = 6) and calcified (n = 6) aortic valves and cultured for 21 days in standard growth medium (control) or chondrogenic medium. Groups were compared by Wilcoxon matched-pairs signed rank test for paired data (control vs chondrogenic medium+) and Mann-Whitney test for unpaired data (healthy vs calcified). Lines in scatter plots represent the median.

To compare the potential of VICs to differentiate down the chondrogenic pathway after 21 days of stimulation with chondrogenic medium, we analyzed the expression of chondrogenic markers: *ACAN* (aggrecan) and *COL2A1* (collagen type II alpha 1 chain)^19,20^. Cells from both healthy and calcified aortic valves had increased expression of *ACAN* and *COL2A1* after stimulation, but without important differences between the groups (Fig. 5). The differentiation of BM-MSCs was used as a positive control of chondrogenic differentiation (Supplementary Fig. S4).

### Differentiated cells from healthy valves have higher lipid accumulation and expression of adipogenic genes

**Figure 6:**
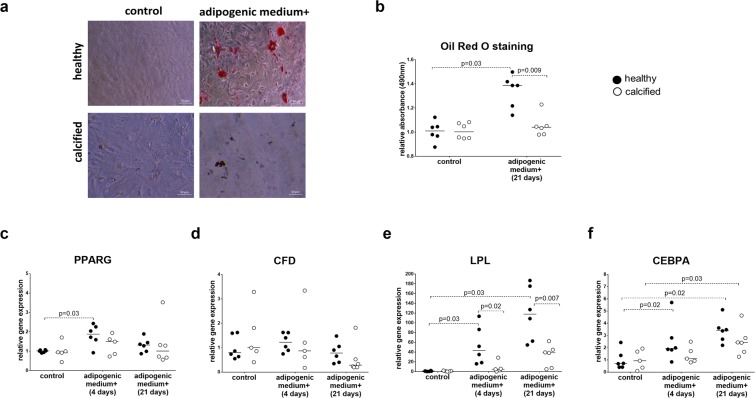
**(a)** Microscopic visualization (20 x objective) of lipid accumulation by Oil Red O staining of interstitial cells isolated from healthy (n = 6) and calcified (n = 6) aortic valves and cultured for 21 days in standard growth medium (control) or adipogenic medium, as indicated. **(b)** Quantification of Oil Red O staining by absorbance at 490 nm. Groups were compared by Wilcoxon matched-pairs signed rank test (control vs adipogenic medium+) or Mann-Whitney test (healthy vs calcified). Lines in scatter plots represent the median. **(c-f)** Relative gene expression, as measured by quantitative reverse transcription PCR, of adipogenic markers: **(c)** *PPARG* (peroxisome proliferator-activated receptor gamma), **(d)** *CFD* (complement factor D), **(e)** *LPL* (lipoprotein lipase) and **(f)** *CEBPA* (CCAAT enhancer binding protein alpha) in interstitial cells isolated from healthy (n = 6) and calcified (n = 5–6) aortic valves and cultured for 4 and 21 days in standard growth medium (control) or adipogenic medium. Groups were compared by Student’s t-test (parametric) or Wilcoxon matched-pairs signed rank test (non-parametric) for paired data (control vs adipogenic medium+) and unpaired Student’s t-test (parametric) or Mann-Whitney test (non-parametric) for unpaired data (healthy vs calcified). Lines in scatter plots represent the median.

To investigate the potential of VICs to differentiate down the adipogenic pathway, we stained VICs from both healthy and calcified valves for lipid droplets after 21 days of stimulation with adipogenic medium. The cells did not accumulate lipids while cultured in standard growth medium (Fig. 6a). After stimulation with adipogenic medium the VICs from healthy valves had significantly higher lipid accumulation compared to cells from calcified valves (Fig. 6a,b), indicating a higher adipogenic differentiation potential. In addition, we assessed the following markers of adipogenic differentiation: *PPARG* (peroxisome proliferator-activated receptor gamma), *CFD* (complement factor D), *LPL* (lipoprotein lipase) and *CEBPA* (CCAAT enhancer binding protein alpha)^19,21^, which were measured after the stimulation of VICs with adipogenic medium for 4 and 21 days (Fig. 6c,d,e,f). Data for adipogenic differentiation were normalized to the control group without differentiation harvested at 4 and 21 days, respectively. Raw data are presented in Supplementary Fig. S5.

After stimulation with adipogenic medium the expression of *PPARG* (4 days) and *LPL* (4 and 21 days) increased only in VICs from healthy valves (Fig. 6c,e), and the expression of *LPL* was significantly higher in cells from healthy valves compared to calcified ones (Fig. 6e). *CFD s*howed a similar profile after stimulation, however, without statistical significance (Fig. 6d). Expression of *CEBPA* was upregulated only in VICs from healthy valves after 4 days of stimulation with adipogenic medium, but in cells from both healthy and calcified valves after 21 days (Fig. 6f). Consequently, VICs from calcified valves have a reduced potential to differentiate down the adipogenic pathway. BM-MSCs were used as a positive control of adipogenic differentiation (Supplementary Fig. S6).

### Cells from healthy valves have more stem cell – like properties

**Figure 7:**
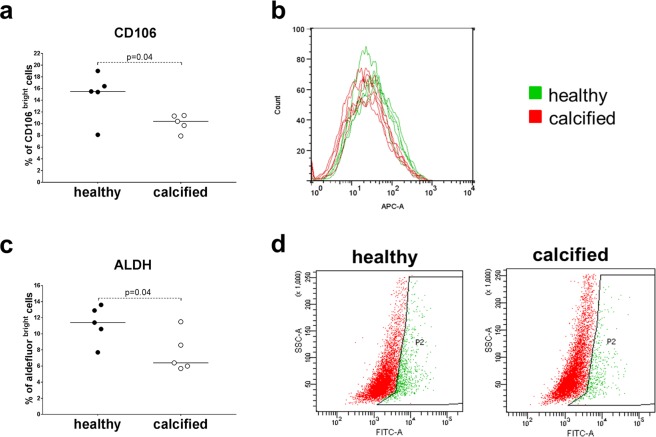
**(a**,**b)** Flow cytometry analysis of surface expression of the mesenchymal stem cell-associated marker CD106 in interstitial cells isolated from healthy (n = 5) or calcified (n = 5) aortic valves. **(a)** The relative percentage of CD106^bright^ cells from healthy or calcified valves are shown as a scatter plot. Groups were compared by Student’s t-test. Lines in scatter plot represent the median. **(b)** Histogram overlays of individual samples from healthy (green curves) or calcified (red curves) aortic valves. **(c**,**d)** Integral aldehyde dehydrogenase (ALDH) activity in interstitial cells isolated from healthy (n = 5) and calcified (n = 5) aortic valves analyzed by flow cytometry following single-cell staining using an enzymatic fluorescent assay. **(c)** The relative percentage of ALDH^bright^ cells from healthy or calcified valves are shown as a scatter plot. Groups were compared by Student’s t-test. Lines in scatter plot represent the median. **(d)** Representative dot plots showing populations of interstitial cells isolated from healthy and calcified valves that are determined as ALDH^dim^ (in red) and ALDH^bright^ (in green).

To investigate whether the differences in differentiation potential between VICs from healthy and calcified valves could occur due to differences in their “stemness” (the degree to which cells possess the functional properties of stem cells^22^), we analyzed the expression of mesenchymal stem cell-associated CD markers: CD146 and CD106 by flow cytometry. As compared to cells from calcified valves, VIC populations from healthy valves contained a more prominent fraction of CD106^bright^ cells (Fig. 7a,b). This could reflect a higher proportion of cells with increased “stemness” in healthy valve samples. CD146 was not expressed in VIC populations.

Further we assessed the other stem cell marker - integral aldehyde dehydrogenase (ALDH) activity in VICs. In a fluorescent enzymatic assay, a specific inhibitor of ALDH, diethylaminobenzaldehyde (DEAB), was used to adjust for background fluorescence (Supplementary Fig. S7). Without treatment with DEAB, VICs from healthy valves contained a larger fraction of cells with high ALDH activity compared to VICs from calcified valves (Fig. 7c,d), again suggesting a higher proportion of stem-like cells in healthy valve samples.

### Cells from healthy valves proliferate faster than cells from calcified valves

**Figure 8:**
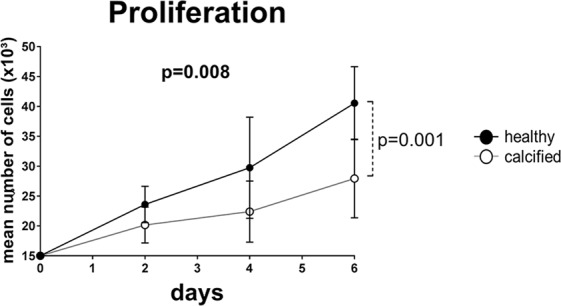
Proliferation of interstitial cells isolated from healthy (n = 5) and calcified (n = 5) aortic valves measured as mean number of cells in triplicates on 0, 2, 4 and 6 days of culturing in growth medium. Data were analyzed by two-way ANOVA with repeated measures. Differences between healthy and calcified donors were determined with Sidak’s multiple comparison post-test and significant p-value is shown on 6^th^ day of measurement. Values are expressed as mean ± SD. Overall p-value from two-way ANOVA is shown in bold.

Before passaging (day 0) and on day 2, 4 and 6 after passaging the cell number from each donor was quantified by direct counting in triplicates. On the sixth day the amount of cells from healthy valves was in 1.5 times higher than from calcified valves (Fig. 8). Overall, cells from healthy valves proliferated faster than cells from calcified valves.

## Discussion

The most important question is which basic cell changes initiate and cause the calcification process. However, it is valuable to elucidate some of the changes induced during the calcification process itself. Our main finding is that cells from healthy valves exhibited higher potential for differentiation into myofibroblastic and adipogenic pathways, higher expression of stem cell-like markers and greater ability to proliferate. In contrast, cells from calcified valves had a more “osteogenic phenotype” with spontaneous calcification and decreased ability to differentiate into other cell types. Possibly not all cells in calcified aortic valves are fully differentiated and there may still be populations of osteogenic precursor cells^23^. Cells from calcified valves also retain their ability to differentiate down the chondrogenic pathway.

Previous comparisons of phenotypes of healthy and calcified aortic valves have mainly been performed on whole-tissue specimens, i.e. pieces of valve leaflets. For example, it was shown that calcified leaflets had changes in extracellular matrix, vascularization, and invasion of inflammatory cells unlike healthy leaflets^3^. In another report, calcified leaflets expressed more TGFβ1, ALP and MMP-9^24^. A microarray study comparing healthy and calcified valves showed differences in expression of genes related to inflammation, calcification and remodeling^25^. Moreover microRNA analysis revealed distinct differences of microRNA profiles between healthy and calcified valves^26^. However, whole valve analysis provided a very heterogeneous picture with a totally different cell population of the valve leaflets including inflammatory cells in calcified valves^27^. We investigated the differences directly in VICs isolated from healthy and calcified valves, and their potential to differentiate into cell types that usually are present in calcified, but not in healthy aortic valves: osteoblasts and myofibroblasts^7^. VICs are the most important cells to compare as they are crucial for the calcification process^7^.

Calcification is the primary endpoint of osteogenic differentiation. Even without stimulation, cells from calcified valves have an osteogenic phenotype, accumulating calcium. However, after stimulation with osteogenic medium, the cells from healthy valves began to calcify, and the cells from calcified valves significantly increased amount of calcified nodules.

Further, we compared the expression of osteogenic genes *BMP2*, *OPG*^15^ and other genes that are involved in the process of calcification *TSP-1*^17^, *POSTN*^16^. In addition we investigated the expression of myofibroblastic genes *ACTA2*, *CNN1* and *TAGLN*^18^. Among these, only *POSTN* was differently expressed between unstimulated control cells from healthy and calcified valves. An interesting finding is that the expression of *POSTN* was inhibited in cells from healthy valves after stimulation with osteogenic medium. Deletion of *POSTN* in a murine model causes aortic valve calcification^16^. This indicates that *POSTN* may play an important role in calcification; a lack of it causes calcification and if calcification is induced then *POSTN* expression is depressed. While the expression of osteogenic genes *BMP2* and *OPG* had similar changes in cells from healthy and calcified valves under osteogenic and myofibroblastic stimuli, the expression of myofibroblastic genes *ACTA2*, *CNN1*, *TAGLN* and other calcification-related marker - *TSP-1* were regulated only in cells from healthy valves. While *ACTA2*, *CNN1*, *TAGLN* and *TSP-1* were upregulated after treatment with myofibroblastic medium, they were downregulated after treatment with osteogenic medium in cells from healthy valves. Probably, myofibroblastic and osteogenic pathways of differentiation are mutually exclusive in the same cell population, as we previously suggested^28^.

After we compared the potential of cells from healthy and calcified aortic valves to differentiate into the chondrogenic pathway. It is known that the process of osteogenesis can occur via the chondrogenic stage where osteoblasts stem from chondrocytes or perichondrial cells (endochondrial bone formation)^29^. Approximately 1% of calcified aortic valves are believed to contain endochondrial bone deposits^30^. Moreover, human calcified valves have higher expression of chondrogenic and osteogenic markers compared to healthy valves^31^. We showed that both the cells from healthy and calcified aortic valves have increased expression of chondrogenic genes *COL2A1* and *ACAN* after stimulation with chondrogenic medium. Probably, as it had been suggested^31^, the chondrogenic pathway as well as the osteogenic pathway is upregulated in VICs to activate osteogenic-like calcification.

To investigate the multipotency of VICs in culture, we differentiated cells from calcified and healthy valves into the adipogenic direction. Adipogenic pathway, unlike osteogenic, chondrogenic and myofibroblastic pathways, is probably not involved in aortic valve calcification and is an independent characteristic of mesenchymal cell multipotency^32^. Higher lipid accumulation and expression of adipogenic gene *LPL*^21^ in cells from healthy valves after stimulation with adipogenic medium indicate that they have higher potential for adipogenic differentiation.

To further assess the stemness of VICs, we compared the expression of selected mesenchymal stem cell markers between VICs from healthy and calcified aortic valves. Mesenchymal stem cells and fibroblasts have been reported to share many of the same stem cell markers^33^. We chose to measure the expression of CD106 and CD146 because they are presented predominantly in mesenchymal stem cells^33,34^. Moreover, CD106 expression correlates with stem cell characteristics such as clonogenic potential and differentiation plasticity^35^. VICs from healthy valves contained a higher fraction of CD106 positive cells. The integral ALDH activity is known to be correlated with “stemness” state of different cell types^36,37^. We have shown that VICs from healthy valves had higher ALDH activity than the cells from calcified valves. Additionally, the cells from healthy valves proliferated faster as compared to calcified ones, which is in agreement with the study by Song *et al*.^38^.

All of these data together could indicate that the VICs from calcified valves are committed to osteogenic and chondrogenic differentiation, and lose their potential to differentiate into other cell lineages. Possibly, normal healthy valves include the population of undifferentiated quiescent VICs^39,40^, as well as bone-marrow stem cells^10,41^, whereas in calcified valves most cells are already partially differentiated into osteoblast- and myofibroblast-like cells^39,40^. Consequently, cells from healthy valves maintain their “stemness” state, whereas stem cell depot in calcified valves may be depleted and the cells are programmed to differentiate only down the the osteo/chondrogenic pathway. The presence of circulating osteogenic precursor cells in patients with calcified valves may stimulate the osteogenic potential of VICs from calcified valves^23^.

Our study has several limitations. Firstly, even though both VIC cultures were isolated and grown in the same conditions and the differences we find after some passages should reflect initial differences in cultures *in vivo*, there might be aberrations which could be avoided by studying VICs right after isolation. Because the number of freshly isolated cells from aortic valves is quite low, and the initial passages are contaminated with mineral debris from the valve tissue, it is not possible with our present methods to study differences in phenotype without proliferation and several passages of the cells. A suitable method here would be, for example, single-cell transcriptomic analysis.

Further, we investigated the potential of VICs isolated from healthy and calcified aortic valves to differentiate down four main pathways: myofibroblastic, osteogenic, chondrogenic, and adipogenic. At the same time, there is a possibility suggested by several authors^42–44^, that all myofibroblastic stage is merely an intermediate stage on the way to osteoblastic differentiation. Our study, being a one-step differentiation study, does not pursue this hypothesis, but the data we obtain does not contradict it. It would be tempting to induce osteoblastic differentiation in healthy VICs and investigate the timeline of this process. However, this requires a substantial amount of healthy cells and lies outside the scope of the current study.

Another factor left out of the current investigation, is the proportion of calcification versus valve fibrosis. While the valves are replaced based on the obstruction they create for the blood flow, the degree of actual calcification may vary and calcification may differ from one leaflet to the next. This has not been taken into account.

Finally, we used cells isolated from valves of both male and female patients. Due to technical reasons and small number of donors, we did not compare cells from male and female patients. Such comparison would be interesting, as the stenotic valves of female patients are characterized by higher level of fibrosis whereas aortic valves of male patients have higher density of calcification^45^.

In conclusion, with the above-mentioned limitations the present study demonstrates that interstitial cells from calcified aortic valves have changed their phenotype and function compared to the healthy cells. The capacity to differentiate into other cell lineages is limited in cultured cells from calcified valves, while the cells from healthy valves have higher multipotent differentiation potential and higher expression of stem cell-associated markers. This difference is retained in the cells proliferated in the laboratory under similar conditions after several (2–6) passages. It is tempting to suggest that the changes in signaling pathways at an early stage of calcification are crucial both to understand and possibly to stop the calcification process. An evolving possibility might be the ability to change phenotype as a therapy^46^.

## Materials and Methods

### Ethical considerations

The project was approved by the Regional Ethics Committee South East Norway and performed in accordance with principles of the Declaration of Helsinki. All patients gave written informed consent. Aortic valves were harvested at the Department of Cardiothoracic Surgery, Oslo University Hospital, Oslo, Norway. Healthy valves were collected from explanted hearts of heart transplant recipients without a history of heart valve disease and with macroscopically normal valves. Calcified aortic valves were harvested from patients undergoing aortic valve replacement. The basic characteristics of the donors used in the experiments are summarized in the Supplementary Table S1.

### Cell isolation and culture

Human VICs were isolated as previously described^47^. Briefly, the excised aortic valve leaflets were treated with 1 mg/ml collagenase II (LS004177, Worthington Biochemical Corporation) for 10 minutes and endothelial cells were scraped off from both sides of the leaflets. Then the leaflets were subjected to digestion with 1 mg/ml collagenase II overnight at 37 °C. The cell suspension was homogenized by pipetting and centrifuged at 300 × g for five minutes. VICs were cultured in standard growth medium (DMEM (41966-052, Gibco) supplemented with 10% FBS (HyClone, SH30070.03, GE Healthcare) and 50 µg/mL of gentamicin (15750-037, Gibco) at 37 °C in 5% CO_2_ until confluence of 70–80% before passaging. Cells from passages 2 to 6 were used for all experiments.

### Osteogenic differentiation

To induce osteogenic differentiation VICs were seeded in 24-well plates (25 × 10^3^ cells per well) in standard growth medium, and after 24 hours the medium was changed to osteogenic medium: standard growth medium supplemented with 50 µM ascorbic acid (A4544, Sigma-Aldrich), 0.1 µM dexamethasone (D4902, Sigma-Aldrich) and 10 mM beta-glycerophosphate (G9422, Sigma-Aldrich). For controls, VICs were cultured in standard growth medium without stimulation. The osteogenic medium and standard growth medium were changed twice a week for 21 days. It is a commonly used timepoint for analysis of calcification and osteogenic genes expression^48–50^. After 21 days all cells were subjected to RNA isolation and RT-qPCR analysis or stained by Alizarin Red to visualize and quantify calcium deposits (please see the Supplementary Methods for protocol of Alizarin Red staining and quantification).

### Myofibroblastic differentiation

To induce myofibroblastic differentiation VICs were seeded in standard growth medium in 24-well plates (25 × 10^3^ cells per well) pre-coated with type I collagen from rat tail (A10483-01, Gibco). After 24 hours the medium was changed to myofibroblastic medium: DMEM supplemented with 1% FBS (HyClone, SH30070.03, GE Healthcare) and 5 ng/mL transforming growth factor beta 1 (TGFβ1) (240-B, R&D Systems). Cells were differentiated for either 4 or 14 days (in the latter case the differentiation medium was changed twice a week) in accordance with other studies^24,51–53^. As a control, VICs were cultured in standard growth medium with 10% FBS for the same time as stimulated cells. After 4 and 14 days cells were collected for immunocytochemistry, flow cytometry, or RT-qPCR.

### Gel contraction

3D collagen gel constructs were created as described previously^54^ (see Supplementary Methods) and contained 5 × 10^4^ VICs in 100 µL of gel per well. Next day gels were detached from the walls of the wells and DMEM with 1% FBS was added with or without (control) 5 ng/mL TGFβ1. Images of detached collagen gels were acquired using a scanner (Epson perfection V700 PHOTO) every 24 hours for seven days. Collagen gel area was measured using Science Linker software and percent contraction was calculated as the change in area from the initial area at time zero.

### Immunocytochemistry

VICs were seeded onto chamber slides pre-coated with collagen I and differentiated into myofibroblasts during 14 days as it was described above. Then the cells were fixed with 4% paraformaldehyde (Sigma-Aldrich, P6148) diluted in PBS for five minutes at room temperature, permeabilized in 0.5% Triton X-100/PBS for five minutes and blocked with 10% FBS/PBS at room temperature for 30 minutes. Then the cells were incubated for one hour with primary antibodies: mouse monoclonal anti-α-SMA (sc-32251, Santa-Cruz) and rabbit polyclonal anti-calponin (ab46794, Abcam) and washed with PBS. The respective secondary antibodies Alexa 647-labeled goat anti-mouse IgG2a (A21241, Invitrogen) and Alexa 488-labeled goat anti-rabbit IgG (H + L) (A11008, Invitrogen) were applied for 40 minutes at room temperature. After washes with PBS, the nuclei were stained with Hoechst (33258, Invitrogen). Then cells were washed with PBS and visualized by epi-fluorescence microscopy (Axio Observer Z1, Zeiss).

### Flow cytometry

Expression of the surface markers CD106 and CD146 was analyzed by flow cytometry. VICs cultured in standard growth medium were washed with PBS, lifted using non-enzymatic cell dissociation solution (C5789, Sigma-Aldrich), resuspended in PBS with 2% FBS and centrifuged for five minutes at 300 × g. The cells were then resuspended in PBS with 2% FBS and 10 mM NaN_3_ with addition of fluorochrome-labeled antibodies: anti-CD106-APC (A15721, Molecular Probes), anti-CD146-FITC (E11-1469-42, eBioscience) and corresponding isotype control antibodies in concentrations according to manufacturer’s recommendations. The cells were incubated for 30 minutes at +4 °C in the dark, then washed with PBS/2% FBS/10 mM NaN_3_ and analyzed in a flow cytometer (FACSCanto II, BD Biosciences) using FACSDiva (BD Biosciences) and FlowJo software.

To investigate the expression of myofibroblastic marker proteins, VICs were stimulated with myofibroblastic medium for 4 or 14 days and stained with antibodies against α-smooth muscle actin - anti-αSMA-FITC (ab8211, Abcam) and calponin - anti-calponin-1-APC (NBP2-47757, NovusBio) or appropriate isotype controls in concentrations recommended by the manufacturer (see Supplementary Methods for protocol of staining). Finally, cells were analyzed by flow cytometry as described above.

In order to investigate the integral activity of aldehyde dehydrogenases (ALDH) in VICs, the cells were stained using a non-immunological fluorescent reagent kit (ALDEFLUOR Kit, 01700, STEMCELL Technologies) and analyzed by flow cytometry as described above. Diethylaminobenzaldehyde (DEAB) treated VIC cultures were used as negative control.

### Chondrogenic differentiation

The cells were transferred to 1 ml eppendorf tubes (50 × 10^4^ cells per tube) and centrifuged at 300 g for five minutes. The resulting cell pellets were statically cultured in DMEM supplemented with 1 mM sodium pyruvate (13360-039, Gibco), 0.1 mM ascorbic acid-2-phosphate (A8960, Sigma-Aldrich), 1% ITS (25 mg/mL insulin, 25 transferrin mg/mL, and 25 ng/mL sodium selenite; I1884, Sigma-Aldrich), 1.25 mg/mL human serum albumin (059487, Octapharma), 500 ng/mL bone morphogenic protein-2 (181720, InductOs) and 10 ng/mL TGFβ1 (240-B, R&D Systems). For controls, the cell pellets were cultured in standard growth medium without stimulation. Chondrogenic medium and standard growth medium were changed twice a week for 21 days in accordance to other studies^10,55^. After 21 days all cells were subjected to RNA isolation and RT-qPCR analysis, few samples were stained with Alcian Blue to detect proteoglycans (please see Supplementary Methods for protocol of staining). For positive control of chondrogenic differentiation, the BM-MSCs were stimulated with standard growth medium and chondrogenic medium.

### Adipogenic differentiation

To induce adipogenic differentiation VICs were seeded on 24-well plates (25 × 10^3^ cells per well) in standard growth medium, and after 24 hours the medium was changed to adipogenic medium (standard growth medium supplemented with 10 µg/ml insulin (I9278, Sigma-Aldrich), 0.5 mM 3-isobutyl-1-methylxanthine (I7018, Sigma-Aldrich), 1 µM dexamethasone (D4902, Sigma-Aldrich) and 1 µM rosiglitazone (R2408, Sigma-Aldrich). Cells were differentiated for either 4 or 21 days (in the latter case the differentiation medium was changed twice a week). As a control, VICs were cultured in standard growth medium with 10% FBS for the same time as stimulated cells. After 4 and 21 days the cells were collected for RNA isolation followed by RT-qPCR analysis. After 21 days cells were also stained with Oil Red O to visualize and quantify lipid droplets in accordance to other studies^10,55^ (please see Supplementary Methods for protocol of Alizarin Red staining and quantification). For positive control of adipogenic differentiation, the BM-MSCs were stimulated with standard growth medium and adipogenic medium.

### Proliferation assay

Cells from donors (n = 3) were passaged in triplicates on 24-well plates (15 × 10^4^ cells per well) in standard growth medium and cultured for 2, 4 or 6 days. At every time point the cells were harvested with trypsin (Trypsin-EDTA solution, 0.05%, T3924, Sigma Aldrich) and centrifuged at 300 x g for five minutes. The amount of cells in every sample was quantified manually using Bürker cell counter.

### Gene expression assay

Total RNA was isolated using Trizol reagent (15596026, Invitrogen,) according to manufacturer’s protocol and quantified by spectrophotometry (NanoDrop ND-1000 Spectrophotometer, Saveen Werner). Reverse transcription was performed using qScript cDNA Synthesis Kit (95047-500, Quanta BioSciences Inc.) according to the manufacturer’s protocol. Quantitative reverse transcription polymerase chain reaction (RT-qPCR) was run using Power SYBR Green detection PCR Master Mix (4367659, Applied Biosystems, Life technologies) and ABI7900 thermal cycler (Applied Biosystems). Primer sequences are presented in Supplementary Table S2. The fold change in gene expression compared to 18 S rRNA was calculated using the comparative ΔΔCT method. All experiments were performed in duplicates.

### Statistical analysis

Statistical analysis was performed using Prism 7 (GraphPad Software). The data regarding Alizarin Red staining were analyzed with Wilcoxon matched-pairs signed rank test (non-parametric) for paired data and Kolmogorov-Smirnov test (non-parametric) for unpaired data. The data regarding RT-qPCR, flow cytometry and Oil Red O staining were analyzed using paired Student’s t-test (parametric) or Wilcoxon matched-pairs signed rank test (non-parametric) for paired data and unpaired Student’s t-test (parametric) or Mann-Whitney test (non-parametric) for unpaired data. Non-parametric tests were chosen in the case of non-equal distribution as determined using Shapiro-Wilk normality test. Data are shown as scatter dot plot, lines represent the median. p ≤ 0.05 was considered statistically significant.

Data from proliferation assay and collagen gel contraction were analyzed by two-way ANOVA with repeated measurements. Differences between healthy and calcified donors were determined with Sidak’s multiple comparison post-test. Values are expressed as mean ± SD. p ≤ 0.05 was considered as statistically significant.

Data of relative gene expression and mean fluorescence intensity (MFI) obtained on cells differentiated into myofibroblasts for 4 and 14 days were normalized to the data obtained on control cells without differentiation harvested on four and 14 days respectively. Data of relative gene expression obtained on cells differentiated down to adipogenic direction were normalized to the data obtained on control cells without differentiation harvested on four and 21 days respectively. Mean values of control group in both cases for both time points were equated to 1.

Indicated n in all experiments are biological replicates of cells from different donors.

## Supplementary information

Supplementary Information

## Data Availability

The datasets generated during and/or analyzed during the current study are available from the corresponding author on reasonable request.
