# Supplementary material for: Interstitial cells in calcified aortic valves have reduced differentiation potential and stem cell-like properties
Source: Sci Rep. 2019 Sep 10;9:12934. doi: 10.1038/s41598-019-49016-0 (PMC6736931; doi:10.1038/s41598-019-49016-0)
Supplement: Supplementary file 1 — Supplementary Information [file 41598_2019_49016_MOESM1_ESM.pdf]

# Supplementary Information

# Interstitial cells in calcified aortic valves have reduced differentiation potential and stem cell-like properties

**Maria Bogdanova[1]*, Arsenii Zabirnyk[1], Anna Malashicheva[2,3,4], Katarina Zihlavnikova Enayati[1], Tommy Alexander Karlsen[5], Mari-Liis Kaljusto[6,7], John-Peder Escobar Kvitting[6], Erik Dissen[1], Gareth John Sullivan[1,5,8,9,10], Anna Kostareva[2,11], Kåre-Olav Stensløkken[1,12], Arkady Rutkovskiy[1,7,13#], Jarle Vaage[7,14#]**

[1] Department of Molecular Medicine, Institute of Basic Medical Sciences, University of Oslo, Oslo, Norway; [2] Almazov National Medical Research Centre, St. Petersburg, Russia; [3] ITMO University, Institute of Translational Medicine, St Petersburg, Russia; [4] St. Petersburg State University, St. Petersburg, Russia; [5] Norwegian Center for Stem Cell Research, Oslo University Hospital Rikshospitalet, Oslo, Norway; [6] Department of Cardiothoracic Surgery, Oslo University Hospital, Oslo, Norway; [7] Institute of Clinical Medicine, University of Oslo, Oslo, Norway; [8] Institute of Immunology, Oslo University Hospital, Oslo, Norway, [9] Hybrid Technology Hub - Centre of Excellence, Institute of Basic Medical Sciences, University of Oslo, Oslo, Norway; [10] Department of Pediatric Research, Oslo University Hospital, Oslo, Norway; [11] Department of Woman and Children Health, Karolinska Institute, Stockholm, Sweden; [12] Center for Heart Failure Research, Oslo University Hospital, Oslo, Norway; [13] Department of Cardiology, Akershus University Hospital, Lørenskog, Norway; [14] Department of Emergency and Critical Care, Oslo University Hospital, Oslo, Norway

[#] Authors contributed equally to this manuscript

***Corresponding author:**

Maria Bogdanova, MSc,

Division of Physiology, Dept. of Molecular Medicine,

Institute of Basic Medical Sciences, University of Oslo,

Postboks 1103 Blindern,

0317 Oslo, Norway

Email: mariia.bogdanova@medisin.uio.no

Phone: +4748621213

## 1. Supplementary Tables and Figures

**Supplementary Table S1**. Details about the donors used in the experiments

| | Source | Number of donors used for experiments in total | Male gender (%) | Age (mean year±SD) |
|---|---|---|---|---|
| **Healthy valves** | Explanted hearts from recipients of heart transplantation without a history of heart valve disease | n=7 | 75 | 54±11 |
| **Calcified valves** | Aortic valves explanted during aortic valve replacement | n=18 | 61 | 76±9 |

**Supplementary Table S2.** Primers used for quantitative PCR.

Names of genes and primer sequences for the SYBR-Green-based quantitative PCR.

| Gene | Description | Forward primer (5'-3') | Reverse primer (5'-3') |
|---|---|---|---|
| *ACTA2* | alpha -smooth muscle actin | CCGACCGAATGCAGAAG | ACAGAGTATTTGCGCTCCGAA |
| *CNN1* | calponin | GCATGTCCTCTGCTCACTTCAA | GGGCCAGCTTGTTCTTAACCT |
| *TAGLN* | transgelin | GGCCAAGGCTCTACTGTCTG | TCTCGATTTTGGACTGCACTT |
| *BMP2* | bone morphogenetic protein 2 | GCCAGCCGAGCCAACAC | CCCACTCGTTTCTGGTAGTTCTTC |
| *OPG* | osteoprotegrin | AAACGGCAACACAGCTCACAAGAA | GCACGCTGTTTTCACAGAGGTCAA |
| *TSP1* | thrombospondin | TCCGCAAAGTGACTGAAGAGAA | TGAACTCCGTTGTGATAGCATAGG |
| *POSTN* | periostin | CCCAGCAGTTTTGCCCATT | TGTGGTGGCTCCCACGAT |
| *PPARG* | peroxisome proliferator activated receptor gamma | AGCCTTCCAACTCCCTCATGGCA | TCCGGAAGAAACCCTTGCATCCT |
| *CFD* | complement factor D | CATGCTCGGCCCTACATGG | CACAGAGTCGTCATCCGTCAC |
| *18 S* | 18S ribosomal RNA | Catalog number: qA-01-0106S (tataabiocenter) | |

Names of genes and Assay ID for the Tagman-based quantitative PCR

| Gene | Description | Assay ID |
|---|---|---|
| *LPL* | lipoprotein lipase | Hs00173425_m1 |
| *CEBPA* | CCAAT enchancer binding protein alpha | Hs00269972_s1 |
| *ACAN* | aggrecan | HS00202971_m1 |
| *COL2A1* | collagen type II alpha 1 chain | Hs00264051_m1 |
| *18S* | 18S ribosomal RNA | Catalog number: 4310893E (Applied Biosystems) |

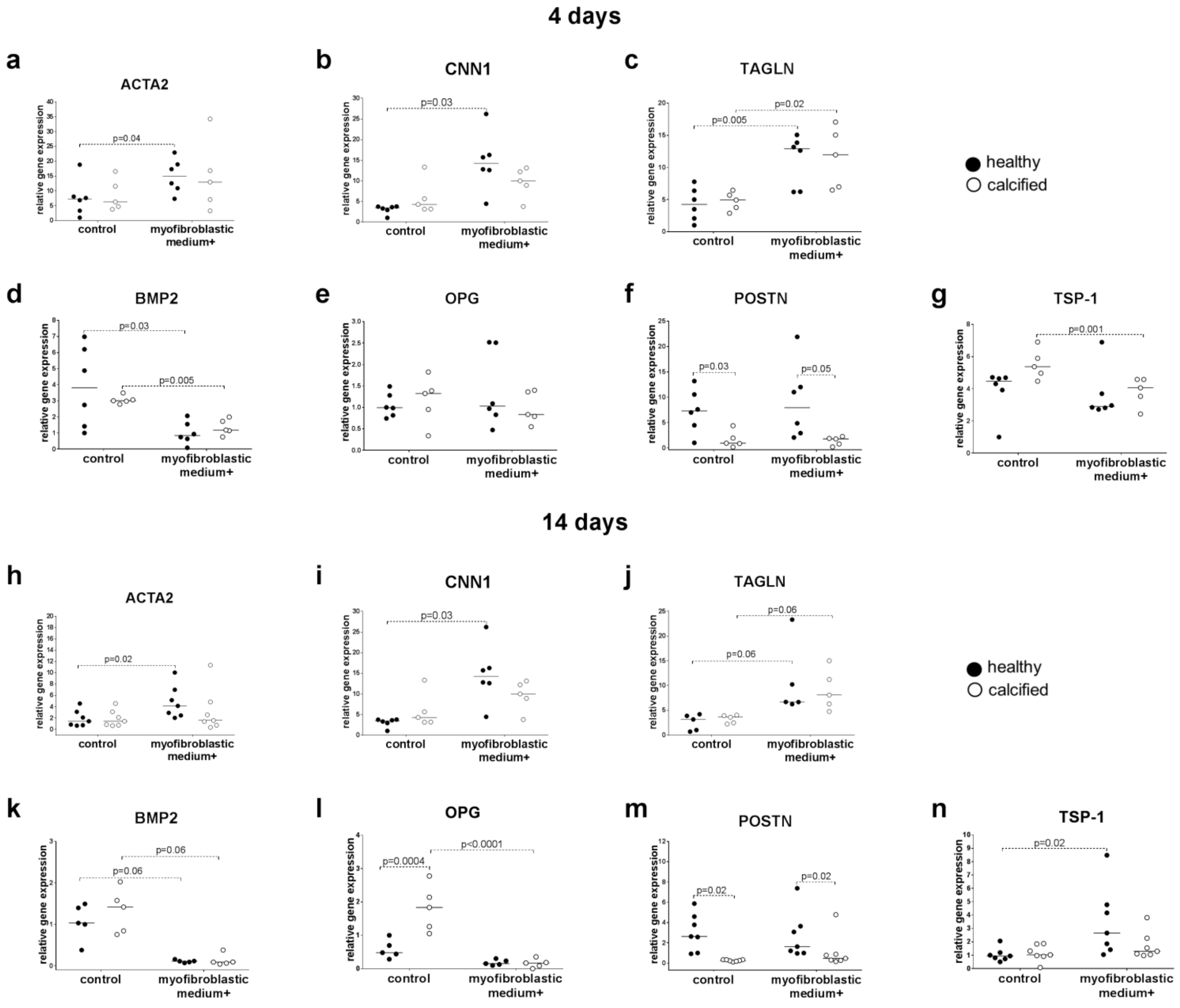


**Supplementary Figure S1**

Relative gene expression, as measured by quantitative PCR, of calcification- and myofibroblast-related genes: **(a, h)** *ACTA2* (alpha–smooth muscle actin 2), **(b, i)** *CNN1* (calponin), **(c, j)** *TAGLN* (transgelin), **(d, k)** *BMP2* (bone morphogenetic protein 2), **(e, l)** *OPG* (osteoprotegrin), **(f, m)** *POSTN* (periostin) and **(g, n)** *TSP-1* (thrombospondin 1) in interstitial cells isolated from healthy (n=6-7) or calcified (n=6-7) aortic valves and cultured for 4 **(a-g)** and 14 (**h-n)** days in standard growth medium (control) or myofibroblastic medium. Groups were compared by Student's t-test (parametric) or Wilcoxon matched-pairs signed rank test (non-parametric) for paired data (control vs osteogenic medium+) and unpaired Student's t-test (parametric) or Mann-Whitney test (non-parametric) for unpaired data **(**healthy vs calcified). Lines in scatter plots represent the median.

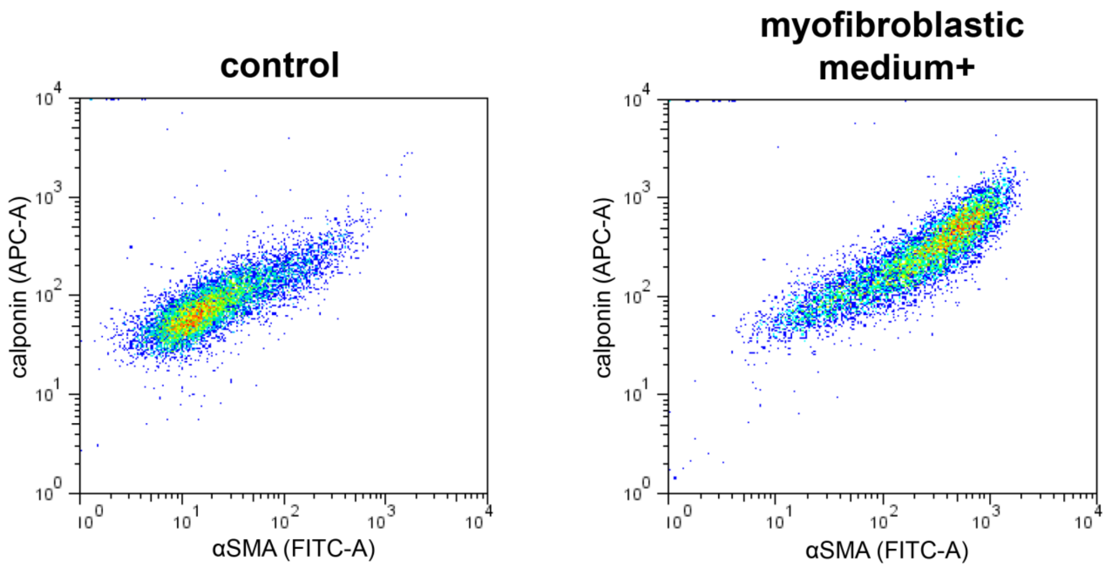


**Supplementary Figure S2**

Simultaneous expression of myofibroblastic markers: alpha-smooth muscle actin (αSMA) (FITC channel) and calponin (APC channel) in interstitial cells isolated from a healthy valve and cultured for 4 days in standard growth medium (control) or myofibroblastic medium. Data were assessed by two-color flow cytometry and shown as a representative density dot plot.

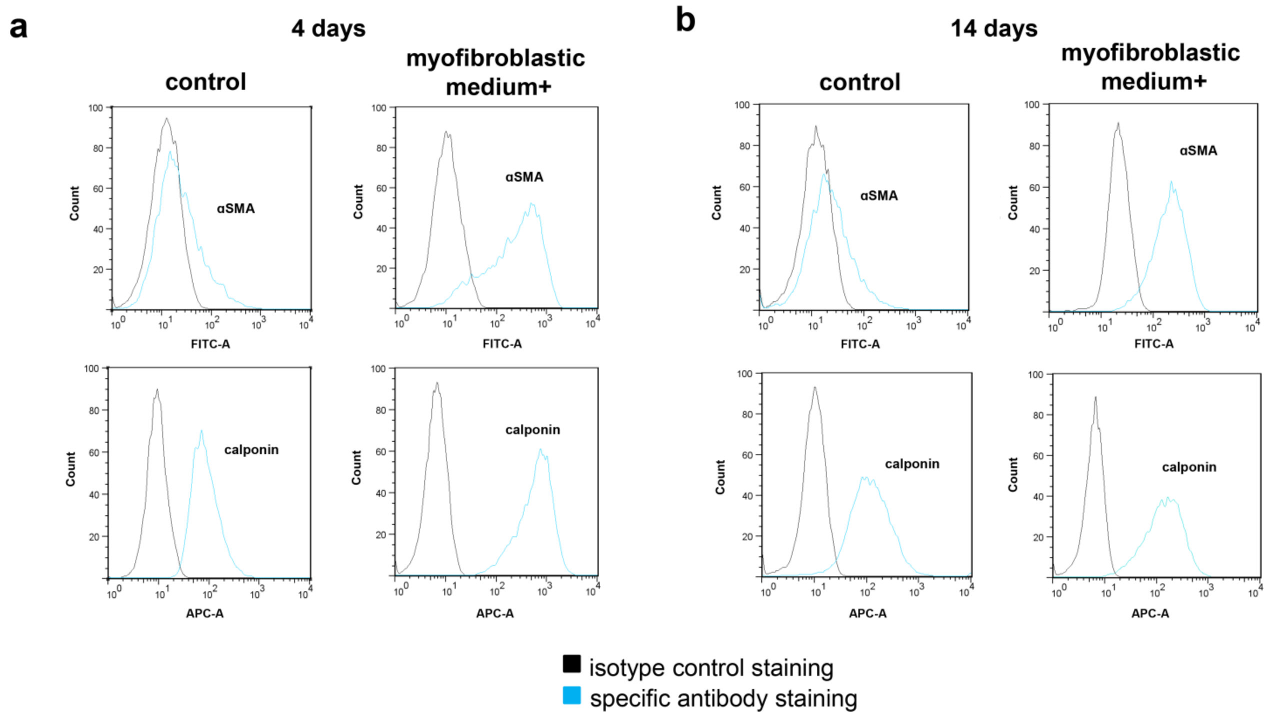


**Supplementary Figure S3**

Representative histogram overlays of individual sample of interstitial cells isolated from a healthy valve, cultured for 4 **(a)** or 14 **(b)** days with standard growth medium (control) or myofibroblastic medium and stained with antibodies for alpha-smooth muscle actin (αSMA) and calponin (blue curves) or isotype control (black curves). Data were assessed by two-color flow cytometry.

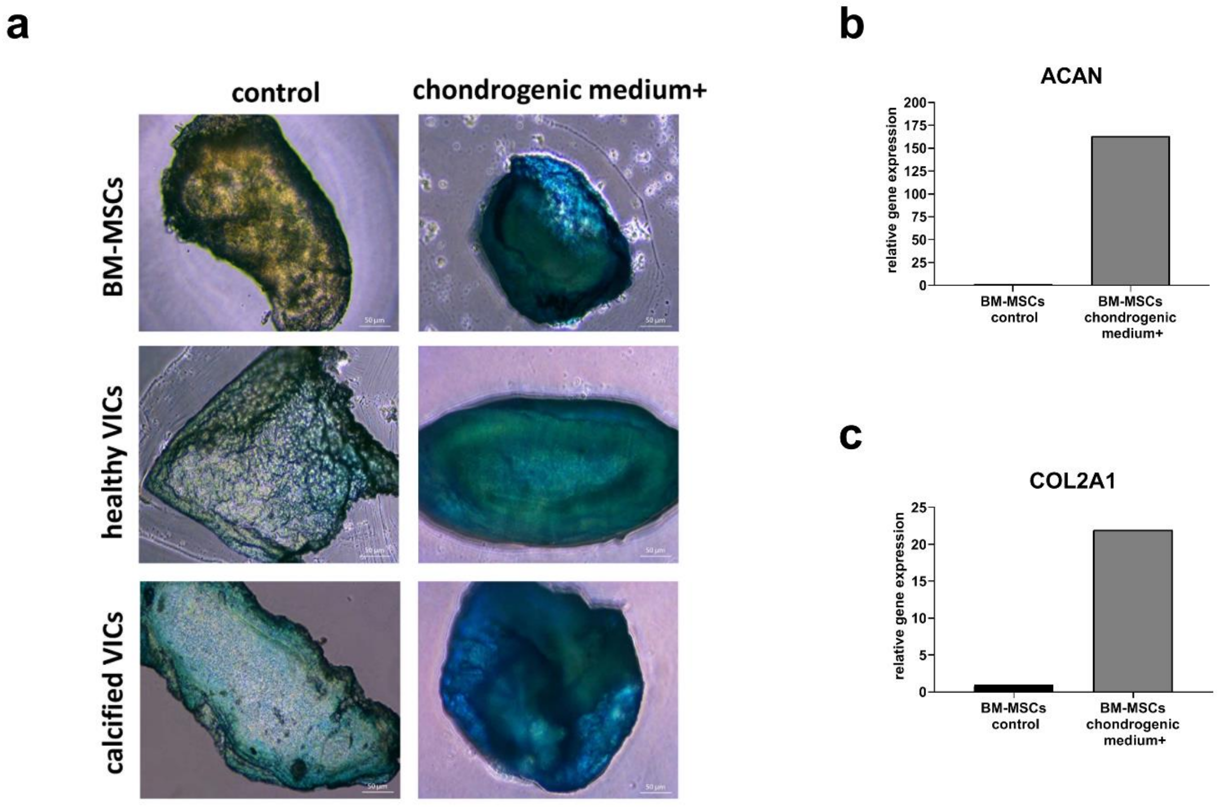


**Supplementary Figure S4**

**(a)** Microscopic visualization (20 x objective) of bone morrow mesenchymal stem cells (BM-MSCs) and valve interstitial cells isolated from healthy and calcified aortic valves that were pelleted, grown in standard growth medium (control) and chondrogenic medium, as indicated, for 21 days and stained with Alcian Blue staining to detect proteoglycans. All types of cells treated with chondrogenic medium were stained an intense dark blue indicating chondrogenic differentiation, whereas control cells were stained light blue. **(b, c)** Relative gene expression, as measured by quantitative reverse transcription PCR, of chondrogenic markers: **(b)** *ACAN* (aggrecan), **(c)** *COL2A1* (collagen type II alpha 1 chain), in BM-MSCs (n=1) cultured for 21 days in standard growth medium (control) or chondrogenic medium.

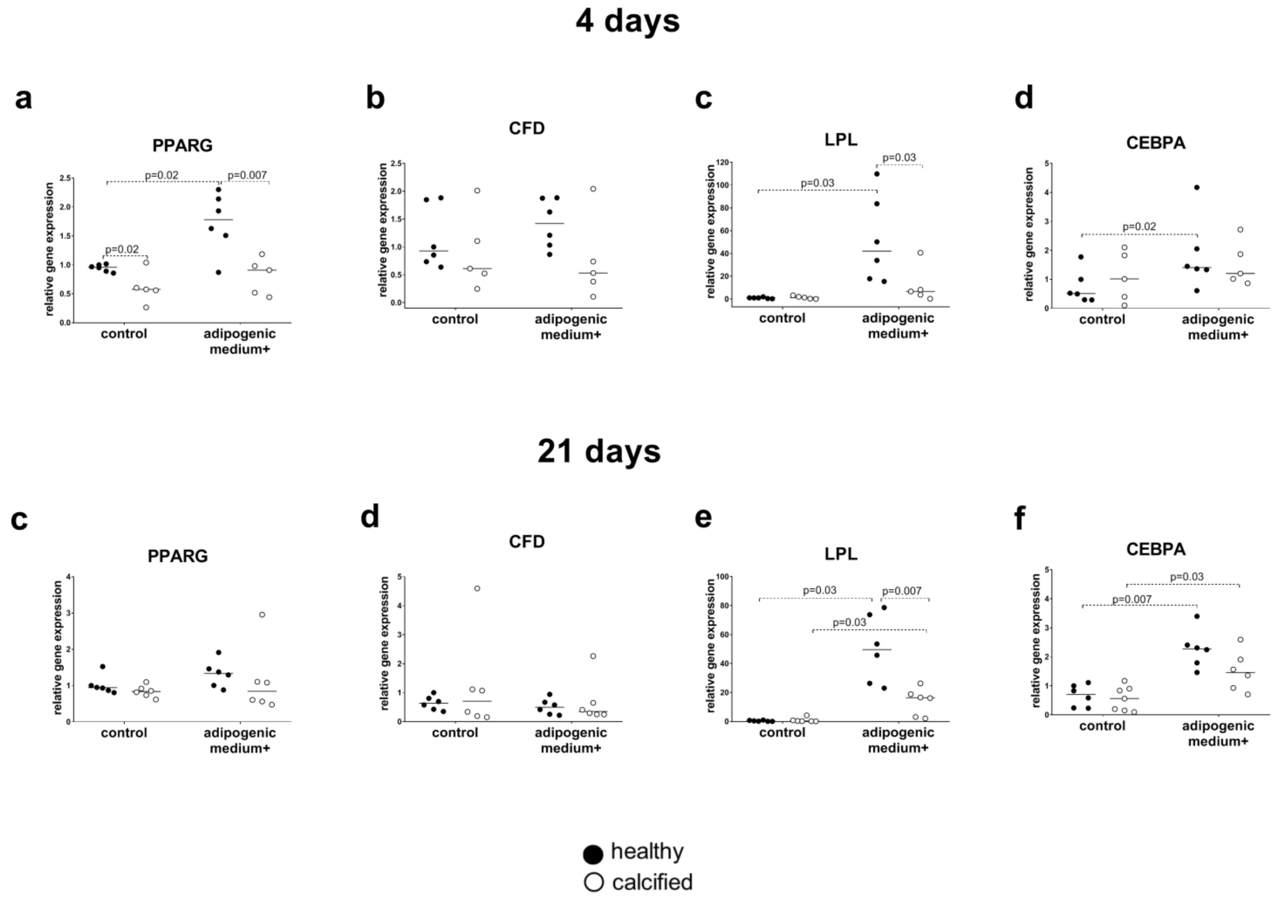


**Supplementary Figure S5**

Relative gene expression, as measured by quantitative PCR, of calcification- and adipogenic-related genes: **(a, c)** *PPARG* (peroxisome proliferator activated receptor gamma), **(b, d)** *CFD* (complement factor D), **(c, e)** *LPL* (lipoprotein lipase), **(d, f)** *CEBPA* (CCAAT enhancer binding protein alpha) in valve interstitial cells isolated from healthy (n=6) or calcified (n=5-6) aortic valves and cultured for 4 **(a-d)** and 21 **(c-f)** days in standard growth medium (control) or in adipogenic medium. Groups were compared by Student's t-test (parametric) or Wilcoxon matched-pairs signed rank test (non-parametric) for paired data (control vs osteogenic medium+) and unpaired Student's t-test (parametric) or Mann-Whitney test (non-parametric) for unpaired data **(**healthy vs calcified). Lines in scatter plots represent the median.

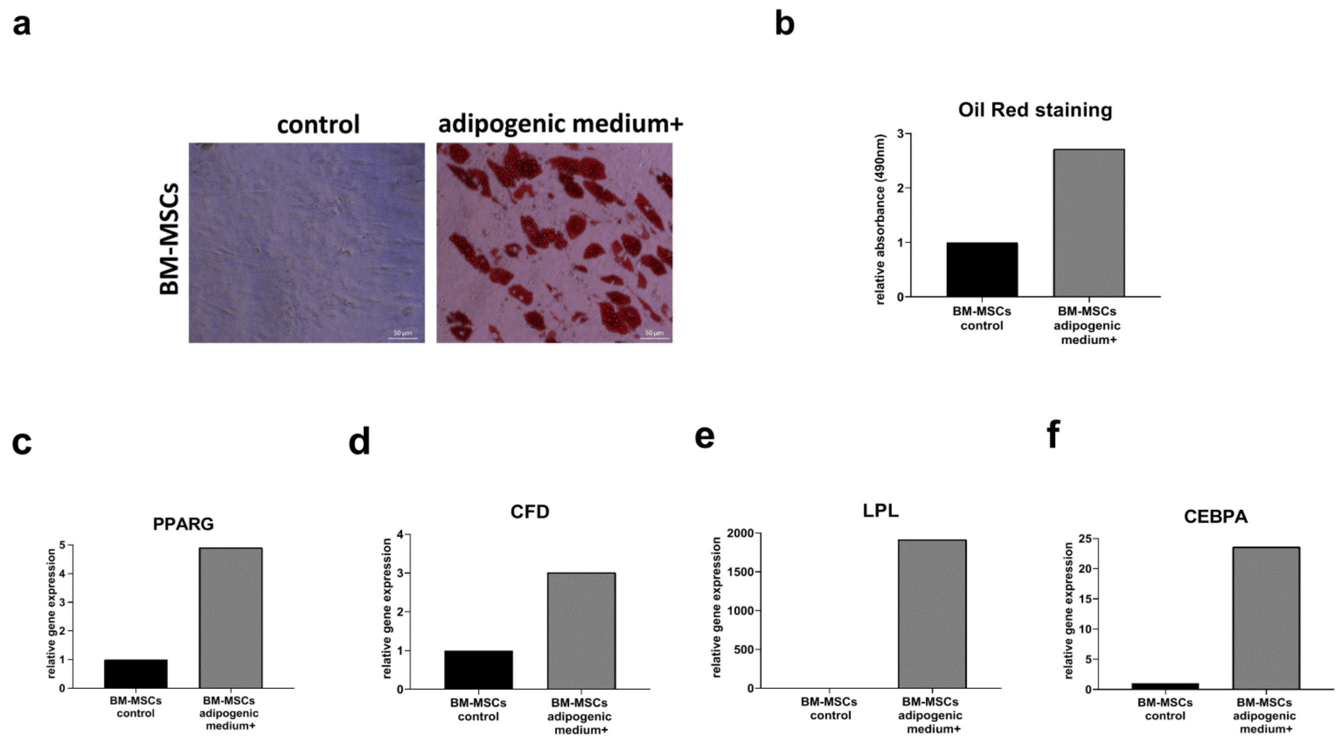


**Supplementary Figure S6**

**(a)** Microscopic visualization (20 x objective) of lipid accumulation by Oil Red O staining of bone morrow mesenchymal stem cells (BM-MSCs) (n=1) cultured for 21 days in standard growth medium (control) or adipogenic medium, as indicated**. (b)** Quantification of Oil Red O staining by absorbance at 490 nm. **(c-f)** Relative gene expression, as measured by quantitative reverse transcription PCR, of adipogenic markers: **(c)** *PPARG* (peroxisome proliferator activated receptor gamma), **(d)** *CFD* (complement factor D), **(e)** *LPL* (lipoprotein lipase and **(f)** *CEBPA* (CCAAT enhancer binding protein alpha) in BM-MSCs cultured for 21 days in standard growth medium (control) or adipogenic medium.

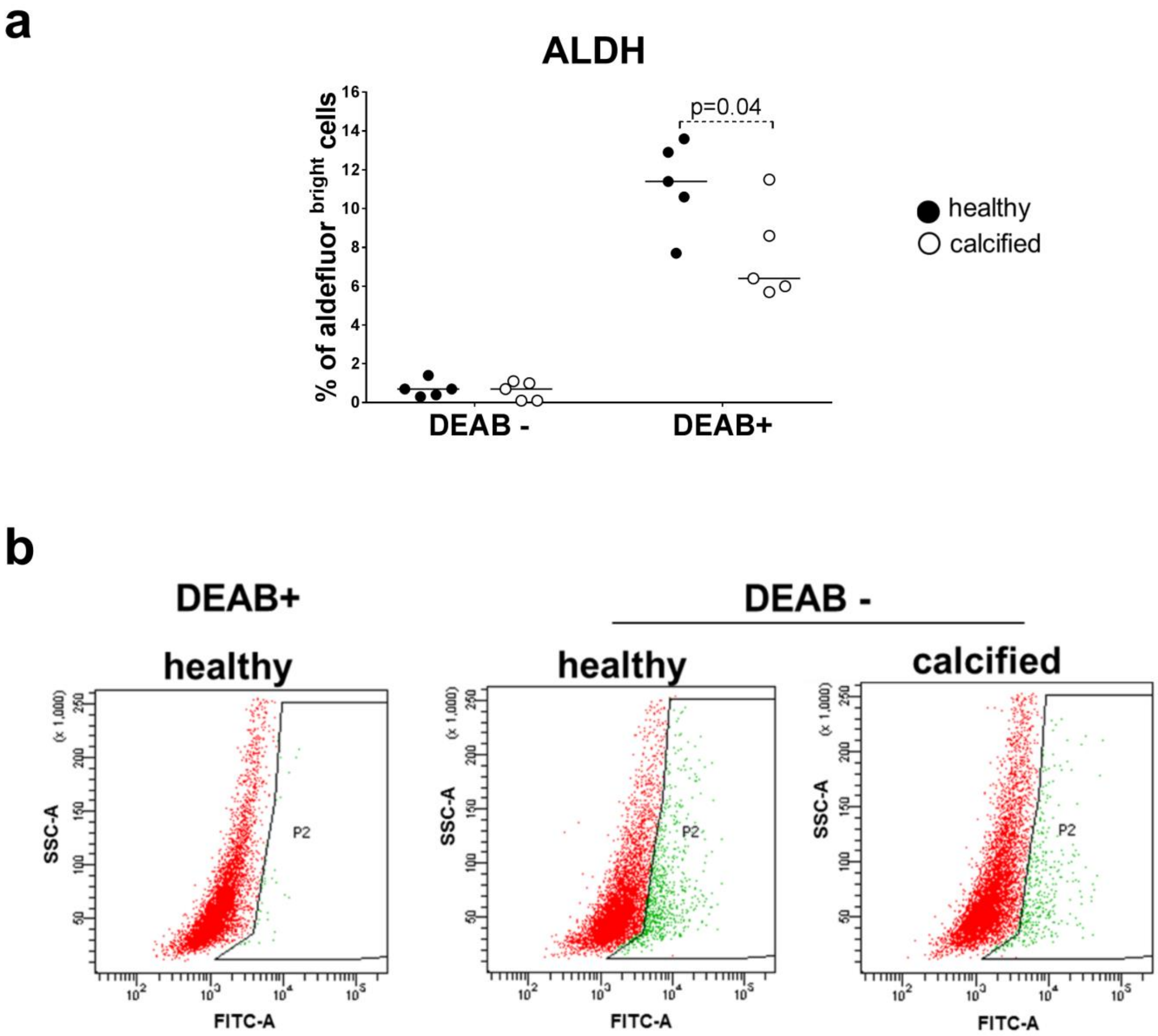


**Supplementary Figure S7**

Integral aldehyde dehydrogenase (ALDH) activity in interstitial cells isolated from healthy and calcified valves analyzed by flow cytometry following single-cell staining using an enzymatic fluorescent assay. A specific inhibitor of ALDH, diethylaminobenzaldehyde (DEAB) was used to control for background fluorescence. **(a)** The relative percentage of $ALDH^{bright}$ cells from healthy or calcified valves is shown as a scatter plot. Lines in scatter plots represent the median. **(b)** Representative dot plots showing populations of interstitial cells isolated from healthy and calcified valves that are determined as $ALDH^{dim}$ (in red) and $ALDH^{bright}$ (in green). The cells treated with DEAB (DEAB+), the cells without treatment (DEAB-).

## 2. Supplementary Methods

**Alizarin staining and quantification**

VICs were washed with PBS and fixed with 70% ethanol for one hour at RT. Then the cells were washed with Milli-Q water and stained with Alizarin Red (A5533, Sigma-Aldrich) according to the manufacturer's instructions. In order to quantify calcification, cells were washed three times with PBS and incubated with shaking in 10% acetic acid for 30 minutes at room temperature. Then cells were detached by cell scraper. Collected cell suspensions were heated to 85˚C for 10 minutes, cooled on the ice for five minutes and neutralized with 1M NaOH. Absorbance was measured at 405 nm using the spectrophotometer (Molecular Devices, USA).

**3D collagen gel constructs**

Collagen gel solution which included 2 mg/mL collagen I (Collagen I, rat tail; A10483-01, Gibco) 5x DMEM (12800-017, Gibco) and 10% FBS (10270-106, Gibco) was neutralized with 0.1 M NaOH and mixed with VICs in 24-well plates (5 x $10^4$ VICs in 100 µL of gel per well). After polymerization of the gel for one hour at 37°C in 5% $CO_2$, 500 µL of standard growth medium was added to each well.

**Staining of cells with antibodies against alpha-smooth muscle actin (αSMA) and calponin for flow cytometry**

Cells were lifted using 0.05% Trypsin-EDTA solution (15400054, Gibco), resuspended in PBS with 2% FBS and centrifuged as described above. After cells were resuspended and centrifuged in PBS and fixed by 1% PFA for 10 minutes at +4ºC. Then cells were washed with PBS/FBS, resuspended in 0.5% saponin (47036, Sigma) (diluted in PBS/2% FBS) and incubated for 20 min at +4ºC for permeabilization. After that the cells were centrifuged and incubated (diluted in saponin/PBS/FBS) for 30 minutes at +4ºC in the dark with anti-αSMA-FITC (ab8211, Abcam), anti-calponin-1-APC (NBP2-47757, NovusBio) or appropriate isotype controls in concentrations recommended by the manufacturer. Finally, cells were washed in saponin/PBS/FBS and analyzed by flow cytometry.

**Alcian Blue staining**

Cell pellets were centrifuged at 400 g for 5 minutes and washed twice with PBS. After the cell pellets were fixed with 4% PFA for 30 minutes and washed first with PBS, they were washed with distilled water and finally with 3% glacial acetic acid solution. Alcian Blue solution (pH 2.5) containing 3% glacial acetic acid and Alcian Blue 8Gx (A-3157, Sigma-Aldrich) was applied to cell pellets for 10 minutes at RT. After the aspiration of Alcian Blue solution the cell pellets were washed once with 3% glacial acetic acid solution and twice with PBS. Dry cell pellets were placed on glass slides and the staining was visualized under the microscope.

**Oil Red staining and quantification**

The VICs were washed with PBS and fixed with 4% PFA for 30 minutes at RT. Then the cells were rinsed with PBS, rinsed again with 60% isopropanol and stained with Oil Red O staining solution (3 parts of Oil Red O (1320-06-5, Sigma Aldrich) mixed with 2 parts of distilled water) for 10 min at RT. The culture was subsequently rinsed four times with distilled water. In order to quantify lipid droplets, Oil Red O staining was measured spectrophotometrically (see Supplementary Methods for details). At first the Oil Red O staining was eluted by cell incubation with 100% isopropanol for 10 min. Absorbance of resulting solution was measured at 490nm using the spectrophotometer (Molecular Devices, USA).
